# Acupuncture and postpartum pyogenic sacroiliitis: a case report

**DOI:** 10.1186/s13256-015-0676-7

**Published:** 2015-09-11

**Authors:** Farida Millwala, Shuo Chen, Vladislav Tsaltskan, Gary Simon

**Affiliations:** Department of Internal Medicine, George Washington University Hospital, 900 23rd St NW, Washington, DC 20037 USA

## Abstract

**Introduction:**

Pyogenic sacroiliitis, a rare form of septic arthritis, occurs in patients following trauma, intravenous drug use, genitourinary infections and pregnancy. Here we report a rare case where both acupuncture and pregnancy served as predisposing risk factors to the development of this infection.

**Case presentation:**

A 33-year-old white woman received several sessions of acupuncture treatment during her gestation at the site of her sacroiliac joint for sciatica; she developed biopsy-confirmed sacroiliitis with methicillin-sensitive *Staphylococcus aureus* during the immediate postpartum period. The diagnosis, medical management and treatment course are described.

**Conclusions:**

Low back and pelvic pain are common conditions during pregnancy and postpartum. Acupuncture is a common modality of medication-free treatment used by many patients. Recognition of the potential complications of such therapies can lead to early diagnosis, accurate treatment, decreased morbidity and increased chances for a successful outcome.

## Introduction

Pyogenic sacroiliitis is a relatively rare form of septic arthritis, accounting for approximately 1% of all cases of septic arthritis [1]. Underlying conditions that may predispose to the development of this infection include trauma, intravenous drug use and genitourinary infections. Pregnancy has also been cited as a potential risk factor for the development of pyogenic sacroiliitis [2]. Here we report a case of postpartum pyogenic sacroiliitis due to *Staphylococcus aureus* in which acupuncture may have provided an additional risk factor for the development of this infection.

## Case presentation

A 33-year-old white woman with no significant past medical history developed right-sided sciatica during the third trimester of her pregnancy for which she received acupuncture on several occasions. One of the puncture locations was located on her back at the level of S2, 5cm to the right of midline, which corresponded to a position directly above her right sacroiliac (SI) joint space. One week after her last acupuncture treatment, she had an uncomplicated vaginal delivery of a healthy infant weighing 4.54kg (10lb). No epidural anesthesia was used. Five days postpartum, she developed rapidly worsening pain over her right buttock to the extent that she was unable to walk and was admitted to our hospital. She denied fever, chills, illicit drug use or trauma to the spine. Her temperature was 37.7 °C, heart rate 90/minute, and blood pressure 121/74mmHg on arrival. A physical examination revealed no swelling or erythema at her hip or spine, but was notable for pain on passive flexion, hyperextension, abduction, adduction, and external and internal rotation of her right hip. There were no other sites of inflammation and no cardiac murmur was heard.

**Fig. 1:**
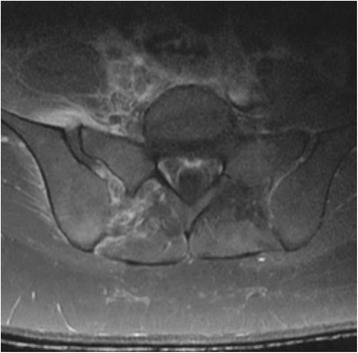
Magnetic resonance imaging of the pelvis showing widening of the patient's right sacroiliac joint space with ‘increased’ fluid in the joint. Also visible are associated inflammatory changes in the surrounding musculature, indicative of an inflammatory process occurring in the joint

Her white blood cell count (WBC) was 13.4, erythrocyte sedimentation rate (ESR) 105, and C-reactive protein (CRP) 192.5. A magnetic resonance imaging (MRI) scan of her lumbosacral spine revealed significant fluid in her right SI joint associated with inflammatory changes extending through the posterior margins of her right iliopsoas musculature and right paraspinal musculature (Fig. 1) in addition to osteomyelitis changes in the adjacent iliac bone and sacrum. During this time, she developed a fever of 39 °C and she underwent a computed tomography (CT)-guided aspiration of her SI joint. Both blood and joint fluid cultures grew methicillin-sensitive *S. aureus.* Urine analysis and urine cultures were unremarkable and an echocardiogram did not reveal any valvular vegetations. She was treated with nafcillin administered intravenously; she had rapid improvement and was discharged home for completion of a 6-week course of antibiotic therapy. Since completion of therapy there has been no evidence of recurrence. Her SI joint tenderness had resolved and her hip pain had improved substantially; she was ambulating independently and laboratory markers of inflammation had dramatically improved.

## Discussion

There is a relatively high incidence of septic sacroiliitis among pregnant women. Whereas septic sacroiliitis represents 1 to 1.5% of all septic arthritides, approximately 10% or more of these infections occur in women during pregnancy or in the postpartum or post-abortion period. In a large series reported by Vyskocil and colleagues, four of 41 (9.7%) women with sacroiliitis were pregnant [3]. In another series, five of 23 women (22%) were in their postpartum period. Almoujahed *et al.* reported cases of 15 women with pregnancy-associated pyogenic sacroiliitis [2] of which six occurred during pregnancy, and six and three, respectively, occurred within 3 weeks of delivery or abortion.

The pathogenesis of septic sacroiliitis is either by direct extension from a local infection [4] or, more commonly, via hematogenous dissemination. Hematogenous development of joint or bone infections tends to occur in joints that, as a result of some local process, are predisposed to infection, for example rheumatoid arthritis. The local damage sets up a site of decreased resistance, a *locus minoris resistantiae*, in which circulating bacteria can settle and begin to proliferate. The frequency of septic sacroiliitis in pregnant women suggests that during pregnancy the SI joint becomes a site of decreased resistance. During the puerperal period there are local factors that may contribute to the predisposition of the SI joint during pregnancy. Minor anatomic changes in the SI joint may occur due to pressures on the SI joint from growth of the gravid uterus or as a result of the trauma of delivery [5]. These changes may affect the microvasculature of the joint leading to microscopic areas of injury on the joint surface making the periosteum more susceptible to bacterial invasion [6].

Acupuncture and other forms of alternative or complementary medicine have become increasingly popular in the USA. Acupuncture is considered relatively safe in pregnancy with few serious adverse consequences [7]. There is a single prior report of sacroiliitis after acupuncture which occurred in a 61-year-old man who had fallen several weeks before the onset of symptoms [8]. In that case, like in ours, the infecting organism was *S. aureus*. Since *S. aureus* can be present on the skin, it is possible that in that case, as well as in our case, the organism was directly inoculated into the joint space by the acupuncture needle. However, this is unlikely. The development of joint symptoms occurred more than 10 days after acupuncture. Direct inoculation of bacteria into the joint space would have led to the more rapid development of symptoms. This delay in the appearance of local symptoms is more supportive of hematogenous spread to a site of minor trauma: the SI joint. Presumably, local trauma resulted in a site of decreased resistance in which bacteria settled during a bout of staphylococcal bacteremia.

While infection of the SI joint is rare, lower back and pelvic pain is very common during pregnancy and the postpartum period. Diagnosing pyogenic sacroiliitis during pregnancy requires a high index of suspicion and requires radiologic procedures [9]. MRI has been shown to be superior to CT, but microbiologic confirmation frequently requires a CT-guided needle aspiration [10]. It is important that the causative organism be identified. Although *S. aureus* is the most common infecting organism, other Gram-negative bacteria such as *Escherichia coli* and *Pseudomonas* species may be involved. Since prolonged antibiotic therapy is necessary, the optimal therapeutic approach is to identify the organism involved and determine its antimicrobial susceptibility.

## Conclusions

This case represents a rare combination of risk factors in which both the patient’s underlying condition of pregnancy and local trauma from acupuncture may have contributed to the development of a relatively rare infectious complication. The history of acupuncture in a pregnant patient led to prompt recognition of this patient’s condition, early treatment and a successful outcome.

## Consent

Written informed consent was obtained from the patient for publication of this case report and accompanying images. A copy of the written consent is available for review by the Editor-in-Chief of this journal.

## References

[1] Hodgson BF. Pyogenic sacroiliac joint infection. Clin Orthop Relat Res. 1989;246:146–9. PubMed. 2766603

[2] Almoujahed MO, Khatib R, Baran J. Pregnancy-associated pyogenic sacroiliitis: case report and review. Infect Dis Obstet Gynecol. 2003;11(1):53–7. Review. PubMed PMID: 12839633; PubMed Central PMCID: PMC1852269. 10.1155/S1064744903000073 PMC1852269 12839633

[3] Vyskocil JJ, McIlroy MA, Brennan TA, Wilson FM. Pyogenic infection of the sacroiliac joint. Case reports and review of the literature. Medicine (Baltimore). Medicine (Baltimore). 1991;70(3):188–97. Review. PubMed. 10.1097/00005792-199105000-00003 2030642

[4] Lau SM, Chou CT, Huang CM. Unilateral sacroiliitis as an unusual complication of acupuncture. Clin Rheumatol. 1998;17(4):357–8. PubMed. 10.1007/BF01451023 9776126

[5] Haq I, Morris V. Post-partum septic sacroiliitis. Rheumatology (Oxford). 2001;40(10):1191–2. PubMed. 10.1093/rheumatology/40.10.1191 11600752

[6] Moros ML, Rodrigo C, Villacampa A, Ruiz J, Lapresta C. Septic shock in pregnancy due to pyogenic sacroiliitis: a case report. J Med Case Rep. 2009;3:6505. doi:10.1186/1752-1947-3-6505. PubMed PMID: 19830110; PubMed Central PMCID: PMC2726500. PMC2726500 19830110

[7] Park J, Sohn Y, White AR, Lee H. The safety of acupuncture during pregnancy: a systematic review. Acupunct Med. 2014;32(3):257–66. doi:10.1136/acupmed-2013-010480. Epub 2014 Feb 19. PubMed PMID: 24554789; PubMed Central PMCID: PMC4112450. PMC4112450 24554789

[8] Tseng YC, Yang YS, Wu YC, Chiu SK, Lin TY, Yeh KM. Infectious sacroiliitis caused by Staphylococcus aureus following acupuncture: a case report. Acupunct Med. 2014;32(1):77–80. doi:10.1136/acupmed-2013-010457. Epub 2013 Oct 17. PubMed PMID: 24135310; PubMed Central PMCID: PMC4151478. PMC4151478 24135310

[9] Oka M, Möttönen T. Septic sacroiliitis. J Rheumatol. 1983;10(3):475–8. PubMed. 6224933

[10] Murphey MD, Wetzel LH, Bramble JM, Levine E, Simpson KM, Lindsley HB. Sacroiliitis: MR imaging findings. Radiology. 1991;180(1):239–44. PubMed. 10.1148/radiology.180.1.2052702 2052702

